# Investigation of temperature effects on wide steel box girder of suspension bridge based on long-term monitoring data

**DOI:** 10.1038/s41598-024-60339-5

**Published:** 2024-04-27

**Authors:** Yanru Wang, Guoliang Zhang, Hui Wang, Lang Liu, Xu Wang, Shuqin Zheng, Jin Huang, Bin Fu, Lei Zhou

**Affiliations:** 1 School of Civil Engineering, Taizhou University, Jiaojiang, 318000 Taizhou China; 2 School of Civil Engineering, Chongqing Jiaotong University, Chongqing, 400041 China; 3 Southwest Technology and Engineering Research Institute, Chongqing, 400041 China; 4 College of Architecture and Energy Engineering, Wenzhou University of Technology, Wenzhou, 150080 China; 5 China Merchants Chongqing Highway Engineering Testing Center Co., LTD, Chongqing, 400074 China

**Keywords:** Temperature effect, Temperature distribution, Structural health monitoring, Steel box girder, Engineering, Materials science

## Abstract

The time-varying temperature distributions on bridge structures may remarkably change structural performance, which may result in differential strain/stress responses on structural members compared with the design conditions. Therefore, it is crucial to have a comprehensive understanding of temperature distributions and its effects on bridges. In this study, taking advantage of structural health monitoring technology, 1-year field monitoring data collected from a long-span suspension bridge were used to investigate the temperature distributions and their effects on the steel box girder. Specifically, the distributions and probability statistics of temperatures on the top and bottom plates were firstly analyzed. Based on which, the transverse and vertical temperature differences on the box girder were further examined, moreover, the representative values of temperature differences for various return periods were calculated by exceedance probability method. At end, a temperature prediction method was proposed to simulated the temperature field distributions during bridge life cycle, to provide substantial temperature data for estimating future operation condition. The results of this study were beneficial to structural evaluation of in-service bridges to ensure their serviceability and integrity in the life cycle.

## Introduction

Large-scale bridges normally suffer from various types of loads, such as highway traffic, railway traffic, wind and thermal effect. Studies have pointed out that temperature effects may have more significant contributions to structural response or structural damage than operational loads^1,2^. Specifically, obvious differential temperatures can always be observed in different components even on the same structural member, due to the changes of solar radiation, environmental temperature and heat conductivity etc.^3^. The variation and non-uniformity of structural temperatures may cause remarkable stress resulting in structural deformation or damage in the long term^4^. This problem tends to be more serious for steel or steel–concrete composite structures that have heat-sensitive features. Therefore, it is crucial to have a comprehensive understanding of temperature distributions and its effects on bridges, no matter for new bridge design or in-service bridge inspection and maintenance.

Numerical simulation as an effective technique in structural analysis is also frequently utilized to simulate environmental temperature field and investigate temperature-induced effects on structural members^5–7^. This research method could provide a fundamental understanding of temperature distributions in bridge site and structural performance under thermal actions in various operational conditions. However, it also has some limitations, for example, temperature distributions are likely to be influenced by many factors, which means they are time-varying and are too complicated to be accurately quantified, and thus structural assessment may turn out to be unreliable.

Attributing to great leaps in sensing, data acquisition and transmission and computing technologies over the past two decades, the application of structural health monitoring (SHM) technology to bridge structures has been widely recognized, to continuously acquire measurement data with respect to structural responses and environmental effects, which is apparently beneficial to bridge condition assessment and maintenance^8–12^. What’s more, the acquired data obtained from the on-structure SHM system have real-time information of environmental status, which provides a reliable approach to account for various load effects imposed on bridge structures, and makes it possible to carefully investigate temperature distribution and temperature effects on structural responses. In fact, a plenty of SHM-based studies have been carried out to clarify this more rational assessment method. For example, thermally induced variations in girder length and mid-span de-flection^13^ and thermal time lags and gradients of the steel box girder^14–18^ were investigated for long-span bridge based on the SHM system, moreover, the temperature distributions of the Tsing Ma Bridge^19,20^ and Benniu Bridge^21^ have been investigated using the monitoring data. In addition, the temperature effects on concrete and steel–concrete bridges were also discussed^22–26^.

These studies have witnessed the temperature-induced strain, internal load, deformation, etc. on various structural members and further affect structural performance in the long term, for which, numerous methods for removing the temperature-induced components from the field measurements or taking them into account in bridge load rating have been developed. However, due to the limited time duration of field measurements, it is difficult to figure out the characteristics of the temperature field during the bridge life cycle as well as the long-term effects on bridge structures, therefore, an effective analysis method that accounts for the whole service life of bridges based on short-term field monitoring data, taking the real temperature distribution and other features, is very needed.

In this study, the transverse and vertical temperature distributions and their probability statistics of a wide steel box girder were examined, based on 1-year field monitoring data acquired by the SHM system installed on the long-span suspension bridge, taking these probabilistic parameters as basis, the representative values for various return periods were calculated by exceedance probability method. At end, a temperature prediction method was proposed by numerical simulation, to provide rational temperature field for estimating future operation conditions. This method was also verified using real monitoring data.

## Long-term monitoring data

### Bridge description

**Figure 1:**
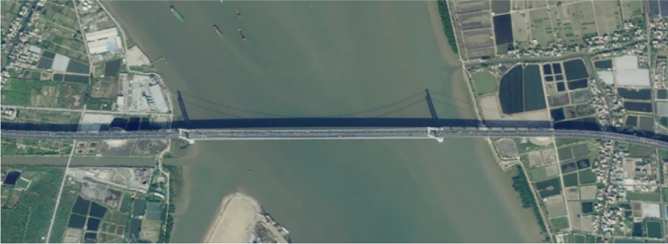
An aerial view of the suspension bridge (image @2023 Google; image by authors).

The case study bridge locates in South China; This suspension bridge has a span arrangement of 360 m + 1200 m + 480 m. The monolithic box girder has a height of 4 m and width of 49.7 m, which was the widest steel box girder in the world at the time when the bridge was built. The wide steel box girder has a rectangular closed box shape, characterized by a wide cross section and a relatively low height. This design enhances its resistance to bending and shear, making it suitable for large-span structures. Regular injuries encompass fatigue cracks in places subjected to high stress, environmental corrosion, localized depression or deformation, and damage to bolted or welded components caused by prolonged tension. The gate-type bridge tower is 193.1 m high, and the main cable is precast steel cable consisting of 169 steel strands while sling composed of 109 parallel steel wires. The suspension bridge had been operational for approximately four years, and an aerial view of the suspension bridge was shown in Fig. 1.

### Structural health monitoring system

**Figure 2:**
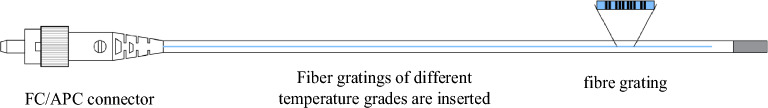
The operation principle of fiber grating temperature sensor.

**Figure 3:**
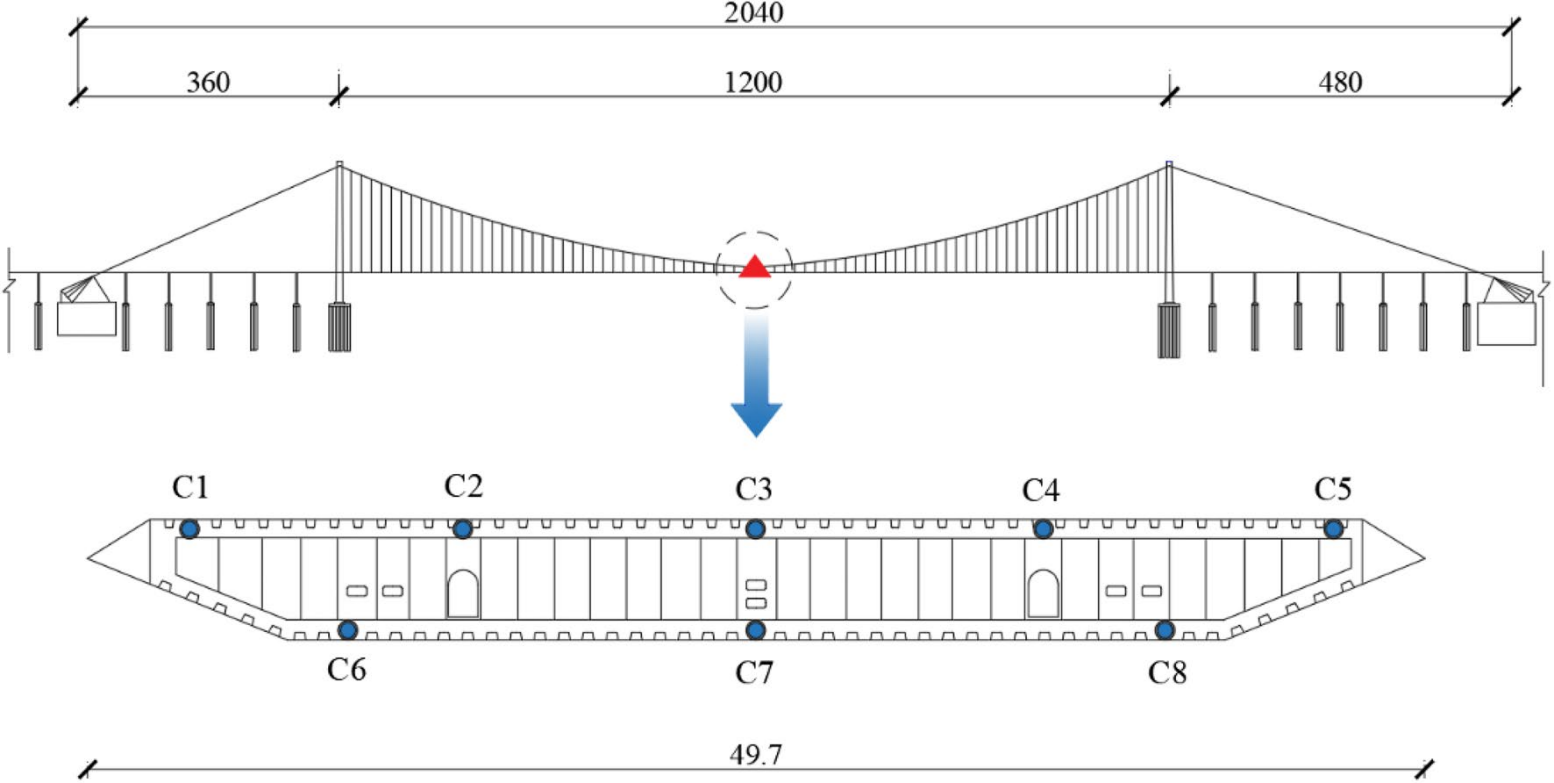
Sensors layout on the box girder.

As an essential measurement for assessing structural behavior and damage, a comprehensive SHM system was installed on this suspension bridge, containing anemograph, accelerometer and temperature sensor etc. The temperature sensor among these is a fiber grating temperature sensor, whose operational mechanism is illustrated in Fig. 2. The refractive index of the fiber grating undergoes changes in response to temperature fluctuations, resulting in a corresponding alteration in the frequency of the light wave. The temperature information can be obtained by monitoring the change in the frequency of light waves. Aiming at monitoring the temperature distribution of the bridge, the box-girder cross-section at the midspan of the main span was selected to install temperature sensors, as shown in Fig. 3. The blue dots represented the measurement points. Five and three measurement points uniformly distributed along transverse direction of the top plate and bottom plate, respectively, these measurement points were marked as C1–C5 and C6–C8 from upstream to downstream of the bridge.

In this study, the monitoring data from January 1, 2021 to December 31, 2021 were used to investigate the temperature distributions and temperature effects on the wide box girder. The sampling frequency of Fiber Bragg grating temperature sensors was 1 Hz and more than 30 million were recorded in total. By initial analysis, it was found the temperature variation within 10 min was negligible, and thus a basic time duration of 10 min was adopted to process the monitoring data to improve computational efficiency, that is, the average temperature within 10 min was the representative value, then 144 temperature samples could be obtained for one day. After scrubbing some bad data, 51,840 observations were obtained as temperature monitoring samples.

### The measured results and characteristic analysis of temperature

#### Daily temperature

Temperature variation would have obvious effects on steel structural members, understanding the variation regularity as well as the probabilistic distribution within a day help with structural deformation estimation. Since the maximum temperature gradient is usually adopted to calculate the temperature-induced stress for steel box-girder design, so the data samples recorded from 00:00 to 24:00 on July 28, 2021, the hottest day of this year, were used to analyze the temperature field. The air temperature of this sunny day was 36–26 °C according to the local weather forecast.

**Figure 4:**
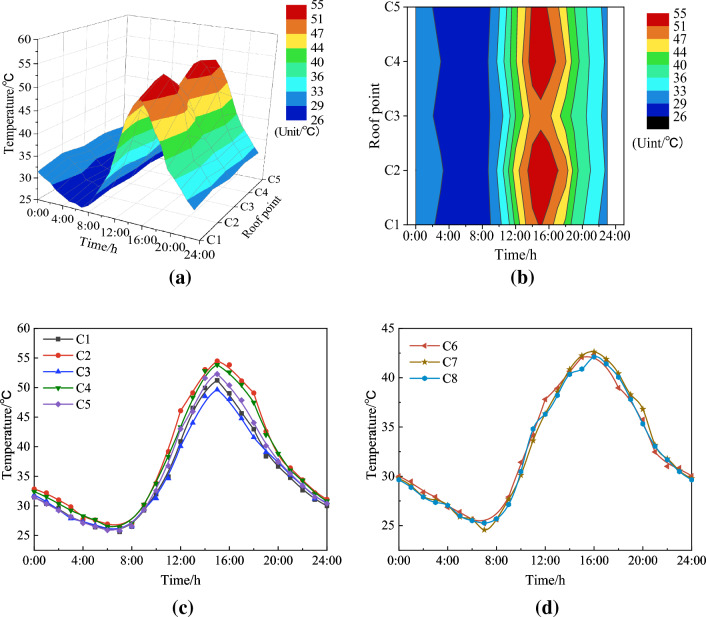
Daily temperature variation of six measurement points. (**a**) 3D image of temperature variation on the top plate; (**b**) projection of the 3D image; (**c**) top plate; (**d**) bottom plate.

Figure 4 displays the temporal variation of temperature at each measurement point on the wide steel box girder for the same day. Observing Fig. 4a,b, the daily temperature of the top plate measurement points C2 and C4 exhibited a faster and higher increase compared to the other measurement points, while measurement point C3 experienced lower temperatures during the same time period. This phenomenon can be attributed to the locations of measurement points C2 and C3. C2 and C3 are situated in the carriageway, where the friction between vehicle tires and the road surface generates heat, causing a quicker temperature rise. Meanwhile, measurement point C3 is positioned in the central barrier. Additionally, Fig. 4c,d reveal that the daily temperature trends of the top and bottom plates are similar, yet there are some distinctions. The minimum and maximum temperatures of the top plate occurred at 7 a.m. and 3 p.m., respectively, with a difference of 28°. In contrast, the maximum temperature of the bottom plate appeared at 4 p.m., exhibiting an 18° difference. This difference arises from the distinct heating mechanisms between the top and bottom plates. The top plate quickly reaches its peak value due to solar radiation, whereas the bottom plate warms up through convection heat transfer in the confined space, resulting in a one-hour delay compared to the top plate. Furthermore, the top plate exhibits a substantial maximum temperature difference of 7.31° among the five measurement points. This considerable temperature variation may lead to significant structural displacement or deformation, necessitating a focus on structural safety.

#### Yearly temperature

**Figure 5:**
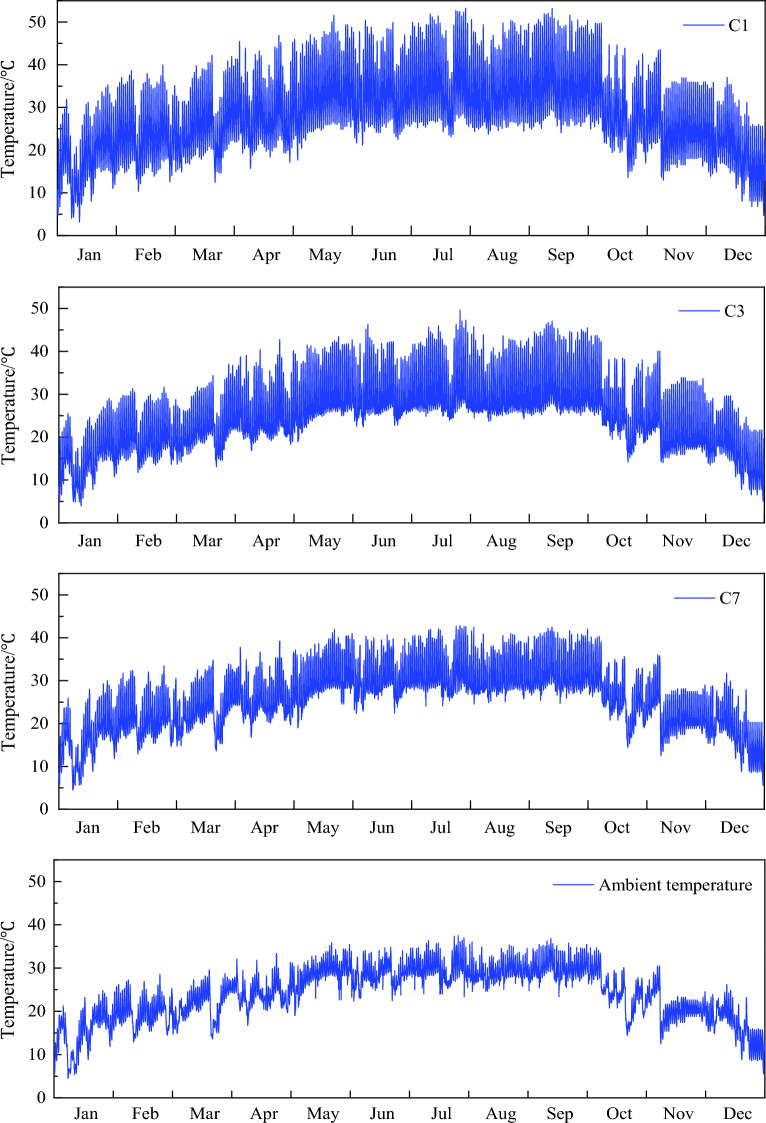
Temperature distribution on the bottom plate.

Temperature distributions in the entire year were also investigated to get the yearly maximum and minimum temperature values as well their differences. The distributions at the measurement points of C1, C3 and C7 were plotted in Fig. 5 as examples, and the extreme values of each measurement point were listed in Table 1. As seen, the highest and lowest temperatures of this steel box-girder in this year were 54.48 °C and 3.01 °C, respectively, and the maximum difference was 51.47 °C, measured at the point C2. Point C3 had smaller variations than other points on the top, while the temperature variations on the top plate were always larger than the bottom plate, due to the solar radiation effect, and the maximum difference between them reached to 11.52 °C.

**Table 1:** Extreme temperature of each measurement point.

Measurement point	C1	C2	C3	C4	C5	C6	C7	C8
Highest temperature/°C	53.21	54.48	49.63	53.84	52.28	42.96	42.79	41.90
Lowest temperature/°C	3.16	3.01	3.98	3.17	3.19	4.36	4.50	4.32
Maximum difference/°C	50.05	51.47	45.65	50.67	49.09	38.60	38.29	37.58

The internal stress resulting from the non-uniform temperature distribution is termed temperature stress. As the temperature stress is positively correlated with the temperature difference, it is essential to analyze the temperature differences among the measurement points in the study of temperature stress. *T*_*i−j*_* (T*_*i−j*_ = *T*_*i*_* − T*_*j*_*, i, j* = 1, 2…8) denotes the temperature difference between distinct measurement points at the same moment. The extreme annual temperature differences at representative measurement points in the transverse and vertical directions of the wide steel box girder are statistically presented in Table 2, leading to the following conclusions: The annual temperature difference in each direction of the cross-section of the wide steel box girder exhibits both positive and negative values, with significantly varying absolute magnitudes. The temperature difference T1–3 at the top plate of the box girder cross-section can reach up to 15.28 °C, signifying that the influence of this temperature difference on the structure is significant and should not be disregarded. The temperature difference between the measurement points of the bottom plate is small, thereby allowing us to disregard its influence on the structure regarding the transverse temperature difference of the bottom plate. The vertical temperature difference between the top and bottom plates (T2–6) can reach up to 21.39 °C, surpassing the upper bound of 20 °C as specified in the Chinese General Specifications for Design of Highway Bridges and Culverts (Ministry of Transport of the People’s Republic of China, 2015)^27^. Hence, a detailed analysis of the temperature field’s characteristics and probability distribution becomes essential.

**Table 2:** Extreme value of the temperature difference at each measurement point.

Temperature difference/°C	*T*_1−2_	*T*_1−3_	*T*_2−3_	*T*_7−6_	*T*_7−8_	*T*_2−6_	*T*_3−7_
Maximum value	5.96	15.28	14.58	1.26	1.38	21.39	10.41
Minimum value	− 9.95	− 3.08	− 2.96	− 0.88	− 1.36	− 5.43	− 7.33

#### Probability statistics of temperature

Histograms of the monitoring temperature data from sensors were plotted to find their probability density functions (PDFs) and statistics. For the general mixture distribution, the parametric PDF in the form of weighted sum of multiple component densities was preferred. In this study, the Gaussian (normal) distribution was adopted as the component density, specifically, the weighted sum of bimodal normal distribution, with its PDF being expressed as

1 $$ f(T) = \gamma_{1} N(T,\mu_{1} ,\sigma_{1} ) + \gamma_{2} N(T,\mu_{2} ,\sigma_{2} ), $$

in which, *T* denoted the observation; $N(T,{\mu }_{i},{\sigma }_{i})$ presents the normal distribution; ${\gamma }_{i}$ was the mixing weight coefficient, satisfying ${\gamma }_{1}+{\gamma }_{2}=1$.

**Figure 6:**
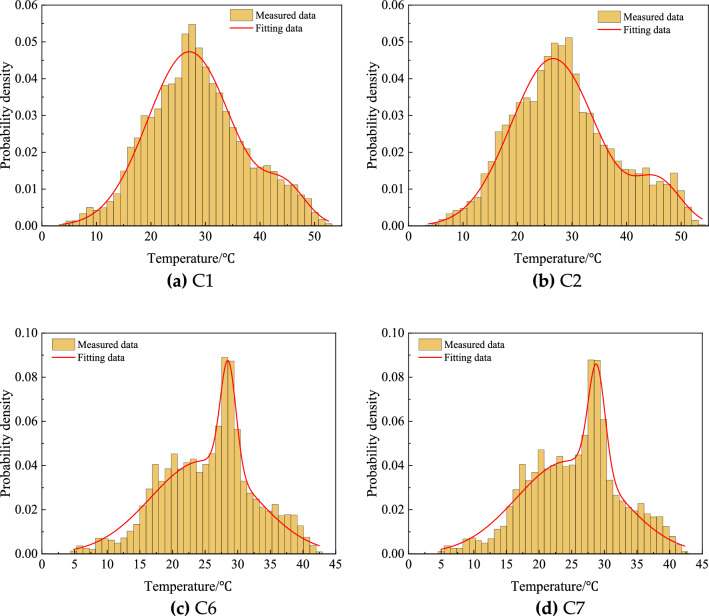
PDFs of measured temperatures.

Then, the initial fitting curves and estimated parameters were verified by ${\chi }^{2}$ test to determine the goodness of fitting. Consequently, the PDFs of the measured temperatures and statistics parameters were displayed in Fig. 6 and Table 3, respectively. It can be seen the fitting curves matched the original data very well and the fitting results can past ${\chi }^{2}$ test at a significance level of 0.1.

**Table 3:** Statistic parameters of measured temperatures.

Measured *T* (°C)	Statistic parameters of the probability density function					
	*γ*_1_	*µ*_1_	*σ*_1_	*γ*_2_	*µ*_2_	*σ*_2_
C1	0.91	27.05	7.67	0.09	44.69	3.81
C2	0.88	26.52	7.72	0.12	45.82	4.18
C3	0.82	20.45	9.01	0.18	26.82	1.32
C4	0.89	26.56	7.81	0.11	46.04	3.96
C5	0.90	27.15	7.54	0.10	44.86	3.90
C6	0.85	20.73	8.06	0.15	28.53	1.21
C7	0.84	20.55	7.98	0.16	28.81	1.29
C8	0.87	20.95	8.09	0.13	28.51	1.16
Environment	0.64	21.06	4.89	0.36	29.12	1.86

**Figure 7:**
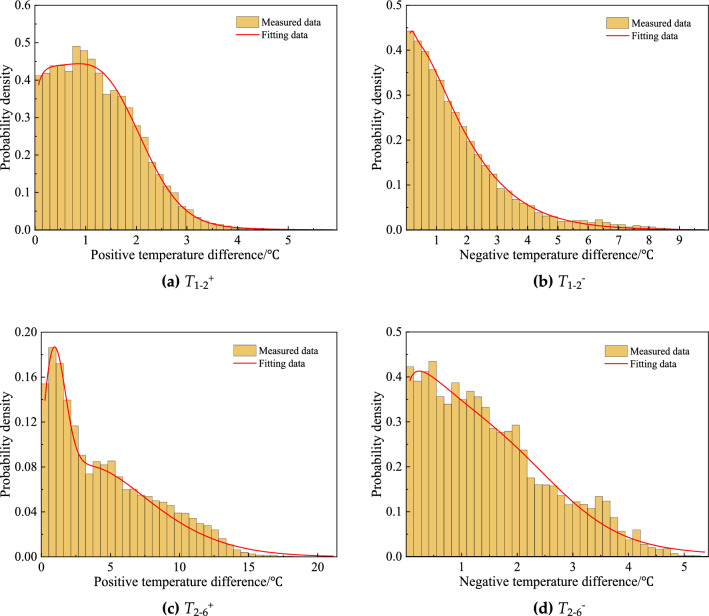
PDFs of measured temperature differences.

The similar analysis was also conducted on the temperature difference of any two measurement points. Regarding the fact that the difference may be positive or negative and whose absolute values varied sharply, the positive and negative differences were separately analyzed in this section. It was found one weighting Weibull distribution plus one weighting normal distribution could approximately describe the probability characteristics of temperature differences. Figure 7 displays the fitted curves representing the typical transverse temperature differences between C1 and C2, as well as the typical longitudinal temperature differences between C2 and C6. Table 4 presents the parameters for the remaining representative measurement points.

**Table 4:** Statistic parameters of measured temperature differences.

Positive/negative temperature difference	Statistic parameters of probability density function					
	*λ*_1_	*a*	*b*	*λ*_2_	*µ*	*σ*
*T*_1−2_^+^	0.45	1.03	1.15	0.55	1.38	0.77
*T*_1−3_^+^	0.44	2.69	1.26	0.56	4.55	1.56
*T*_2−3_^+^	0.74	4.53	1.34	0.26	5.06	0.74
*T*_2−6_^+^	0.70	6.58	1.57	0.30	0.80	0.88
*T*_1−2_^−^	0.97	1.79	1.18	0.03	0.03	0.18
*T*_1−3_^−^	0.82	0.55	1.05	0.18	1.02	0.52
*T*_2−3_^−^	0.72	0.60	1.32	0.28	1.86	0.28
*T*_2−6_^−^	0.70	1.50	1.11	0.30	1.76	1.09

As discussed above, notable temperature differences existed between measurement points, which would induce structural stress in the box girder. The vertical temperature difference was generally considered in bridge design codes, for example, a temperature gradient of 20 °C for a 50-year return period was specified in the Chinese bridge design code, but no recommendation for transverse difference. For a wide steel box girder, the transverse temperature difference may be too large to neglect, therefore, the representative values for various return periods were calculated by the exceedance probability method, as expressed in Eq. (2).

2 $$ P = 1 - P(T\max < T) = 1 - F(T_{\max } ) = \mathop \smallint \limits_{{T_{max} }}^{ + \infty } f(T)dT = \frac{1}{\lambda N}, $$

where *T*_*max*_ was the representative value of temperature difference; *f(T)* was the PDF of *T*; $\lambda$ was the return period and *N* was the number of samples.

As a consequence, the representative values of temperature differences (positive or negative) between any two measurement points were listed in Table 5, with various return periods from ten years to 100 years. Noted that transverse and vertical temperature differences for 50-year return period were 19.55 °C and 23.10 °C, respectively, compared with temperature gradients specified in bridge design codes, the vertical difference was larger than that of 20.0 °C in Chinese *JTG D60*^27^ and 22.0 °C in *AASHTO LRFD*^28^, but closed to that of 24.0 °C in British *BS5400-4*^29^. Moreover, a transverse difference of 19.55 °C may cause significant temperature-induced stress on structural members, and this effect needed to be clarified for special structure design in tropical zones.

**Table 5:** Representative values of temperature difference for various return periods.

Return period/a	*T*_1−2_^+^	*T*_1−3_^+^	*T*_2−3_^+^	*T*_2−6_^+^	*T*_1−2_^−^	*T*_1−3_^−^	*T*_2−3_^−^	*T*_2−6_^−^
10	8.01	17.46	16.86	20.34	10.02	3.58	3.23	4.54
20	8.47	18.36	17.25	21.55	10.74	3.90	3.58	5.29
30	8.73	18.89	17.65	22.24	11.16	4.19	3.88	5.72
40	8.92	19.26	17.97	22.72	11.46	4.42	4.05	6.03
50	9.06	19.55	18.36	23.10	11.79	4.59	4.19	6.27
60	9.15	19.78	18.65	23.42	12.02	4.72	4.33	6.51
70	9.25	20.03	18.90	23.76	12.25	4.81	4.41	6.72
80	9.33	20.22	19.07	24.01	12.48	4.88	4.49	6.84
90	9.42	20.31	19.22	24.12	12.68	4.97	4.54	6.96
100	9.51	20.42	19.38	24.24	12.80	5.05	4.56	7.01

## Temperature field simulation for bridge life cycle

Temperature field analysis based on the monitoring data in 2021 had validated that temperature-induced stress was critical to long-span suspension bridge, and thus understanding temperature field distributions during the bridge life cycle fundamentally help with evaluating the safe state of bridges. However, collecting data for a very long time duration was costly and impractical, and it was impossible to discuss this important issue for structures by real measured data. To solve this problem, a temperature prediction method was proposed in this study, taking advantage of short-term monitoring data and numerical simulation, to provide substantial temperature data for estimating future operation condition.

According to the statistical analysis of temperature data in the previous sections, it was found the PDFs of temperature as well as temperature difference could be described by mixed multimodal Gaussian distribution. Taking the statistic characteristics as a foundation, then the simulation method was developed based on inverse transformation method and Latin hypercube sampler method. The following steps illuminated the detailed process:

1. Set the measurement point C2 as the benchmark, taking the PDF of the measured temperature samples *f(T*_*2*_*)*, to generate temperature samples *T*_*2,all*_ for the whole life cycle, and these samples were further modified by the daily and yearly variation regularity of *T*_*2*_.
2. Utilizing the PDFs of temperature difference *T*_*1−2*_, *T*_*3−2*_ and *T*_*6−2*_ to generate their counterpart samples for the bridge lifetime, denoted as *T*_*1−2,all*_, *T*_*3−2,all*_ and *T*_*6−2,all*_ as similar.
3. By combing the temperature difference equation *T*_*i−2,all*_ = *T*_*i,all*_ − *T*_*2,all*_* (i* = 1, 3, 6) and generated samples *T*_*2,all*_ from step (1), life-cycle temperature samples *T*_*1,all*_, *T*_*3,all*_ and *T*_*6,all*_ for measurement points C1, C3 and C6 could be obtained, and the similar modification in step (1) were also applied to them.
4. To verify the simulation results by comparing the PDFs of *T*_*1,all*_, *T*_*3,all*_ and *T*_*6,all*_ with those of *T*_*2*_, *T*_*3*_ and *T*_*6*_.

### Simulation of temperature sample sequences for the benchmark point C2

The Latin hypercube sampling method is employed to generate a complete life cycle temperature sample, denoted as T2, at the benchmark point C2 of a wide steel box girder. The specific steps are outlined below:

#### To determine the maximum and minimum values of T_2,all_

Set a basic time duration of 10 min for temperature samples, then *n* = 6 × 24 × 365 = 52,560 samples could be obtained for a year, and there were *N* = *100n* samples for bridge life cycle of 100 years. Let the representative return period of temperatures be 50 years and *T*_*2,all,max*_ and *T*_*2,all,min*_ denote the maximum and minimum values of *T*_*2,all*_. The exceedance probability of the two, *P*_*max*_ and *P*_*min*_ were equal to *2/N*, then *T*_*2,all,max*_ and *T*_*2,all,min*_ could be solved by Eqs. (3) and (4).

3 $$ P_{\max } = 1 - F(T_{{2,{\text{all}},\max }} ) = 1 - \int\limits_{ - \infty }^{{T_{{2,{\text{all}},\max }} }} {f(T_{2} )} dT_{2} , $$

4 $$ P_{\min } = F(T_{{2,{\text{all}},\min }} ) = \int \limits_{ - \infty }^{{T_{{2,{\text{all}},\min }} }} {f(T_{2} )} dT_{2} , $$

where, *F(T*_*2*_*)* is the probability distribution function of *T*_*2*_; *f(T*_*2*_*)* is the probability density function of *T*_*2*_.

#### To determine temperature sample sequences

Sampling *T*_*2,all*_ in the interval [*T*_*2,all,min,*_* T*_*2,all,max*_] based on the PDF *f(T*_*2*_*)*, then divided this interval into *M* subintervals with an incremental of $\delta T$, which was determined by Eq. (5).

5 $$ \delta T = \frac{{T_{{2,{\text{all}},\max }} - T_{{2,{\text{all}},\min }} }}{M}. $$

Thereby, the *i*th interval was [(*i*-1) × $\delta T$+*T*_*2,all,min*_, *i* × $\delta T$+*T*_*2,all,max*_], if there were *N*_*i*_ temperature samples in each subinterval, then

6 $$ N_{i} = \left\lfloor {(N - 4)\mathop \int \limits_{{(i - 1) \times \delta T + T_{2,all,\min } }}^{{i \times \delta T + T_{2all,\min } }} f(T_{2} )dT_{2} } \right\rfloor . $$

Noted that *N*_*i*_ made round down in Eq. (6), leading to less number of samples than requirement, while the difference $\Delta N=\left(N-4\right)-\sum_{i=1}^{M}{N}_{i}$. Assuming ${\Delta N}_{i}$ samples were randomly allocated to the *i*th subinterval, then the total number of samples in the *i*th subinterval were $\overline{{N}_{i}}={N}_{i}+{\Delta N}_{i}$.

When *M* was considerably large, the PDF in each interval approximated, to generate uniformly distributed random number in (0,1) by Rand $(\overline{{N}_{i}},\mathrm{0,1}$), then the *i*th interval became (*i*-1) ×  $\delta T$ +*T*_*2,all,min*_ + $\delta T$×rand $(\overline{{N}_{i}},\mathrm{0,1}$). By repeating this process for each subinterval, temperature sample sequences for the bridge life cycle were obtained.

**Figure 8:**
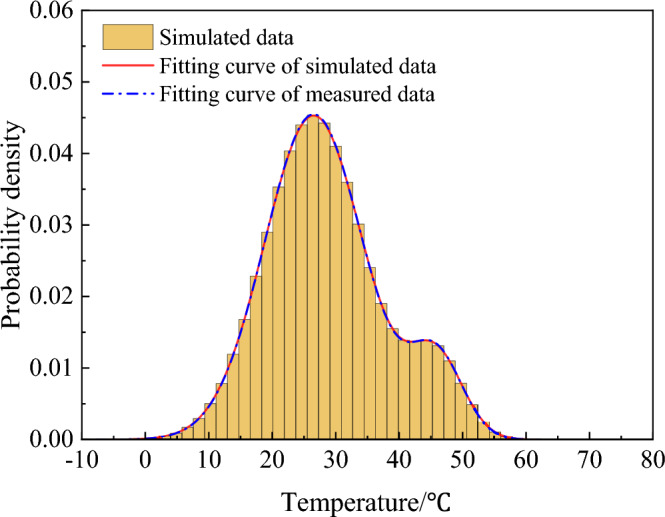
PDF of simulated sample sequence.

**Figure 9:**
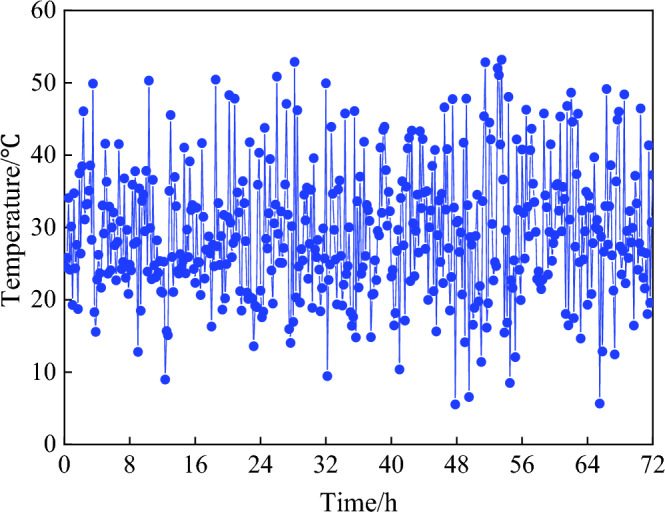
Time history of 72-h simulated temperature.

As a consequence, the simulated sample sequence *T*_*2,all*_ and the PDF were plotted in Fig. 8, it was observed they matched very well and basically validated this simulated method. However, when randomly picked out 72-h temperature data from *T*_*2,all*_ to plot the time history as Fig. 9, it can be seen the variation tendency of simulated temperature samples did not present the periodical regularity like the monitoring data, therefore, further steps were needed to modify the simulation method.

### Modification of the simulated temperature samples

#### Daily characteristics

As shown in Fig. 4, the time history curve of monitoring temperatures appeared as sine function, thus it was reasonable to perform the variation characteristics of daily temperature data by adopting sine function, expressed as

7 $$ T = A\sin (\omega t) + B, $$

in which, *T* was the temperature; *A*, *ω* and *B* were amplitude, frequency and related coefficient, respectively. Which were calculated by $\omega =\frac{2\pi }{24\times 6}=0.0436$, $A=-\frac{{T}_{peak}-{T}_{trough}}{2}$ and $B=\frac{{T}_{peak}+{T}_{trough}}{2}$.

**Figure 10:**
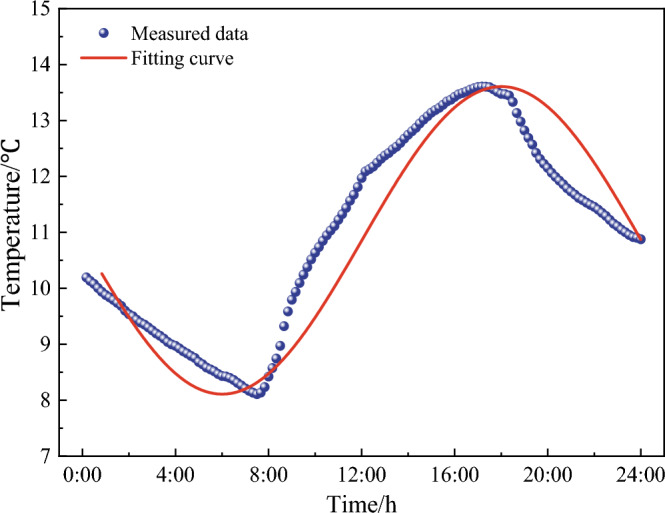
Comparison of simulated data with 24-h observations.

By implementing Eq. (7), the fitting curve of the simulated temperatures for 1 day were compared with the observations in January 1, 2021 in Fig. 10. As seen, they had very similar variation regularity, indicating the simulation accuracy was remarkably improved.

#### Yearly characteristics

On the other hand, periodical (monthly) change was also observed in yearly temperature data, as discussed in the previous sections. Therefore, similar approach could be taken for the simulated yearly data. Specifically, parameters in Eq. (7) were replaced with monthly parameters, for the reality that the temperature was low in January and December and high in July and August, one and a half periodic sin function was considered herein. Besides, there were four extreme temperature values in a month, namely, the maximum peak and trough and minimum peak and trough, denoted as *T*_*max,peak*_, *T*_*max,trough*_, *T*_*min,peak*_ and *T*_*min,through*_, respectively. As a result, the Eq. (7) evolved into

8 $$ T_{m} = A_{m} \sin (\omega_{m} t) + B_{m} , $$

where $$w_{m} = \frac{\pi }{12} = 0.262$$, $$A_{m} = T_{m,peak} - T_{m,trough}$$ and $$B_{m} = T_{m,trough}$$.

**Figure 11:**
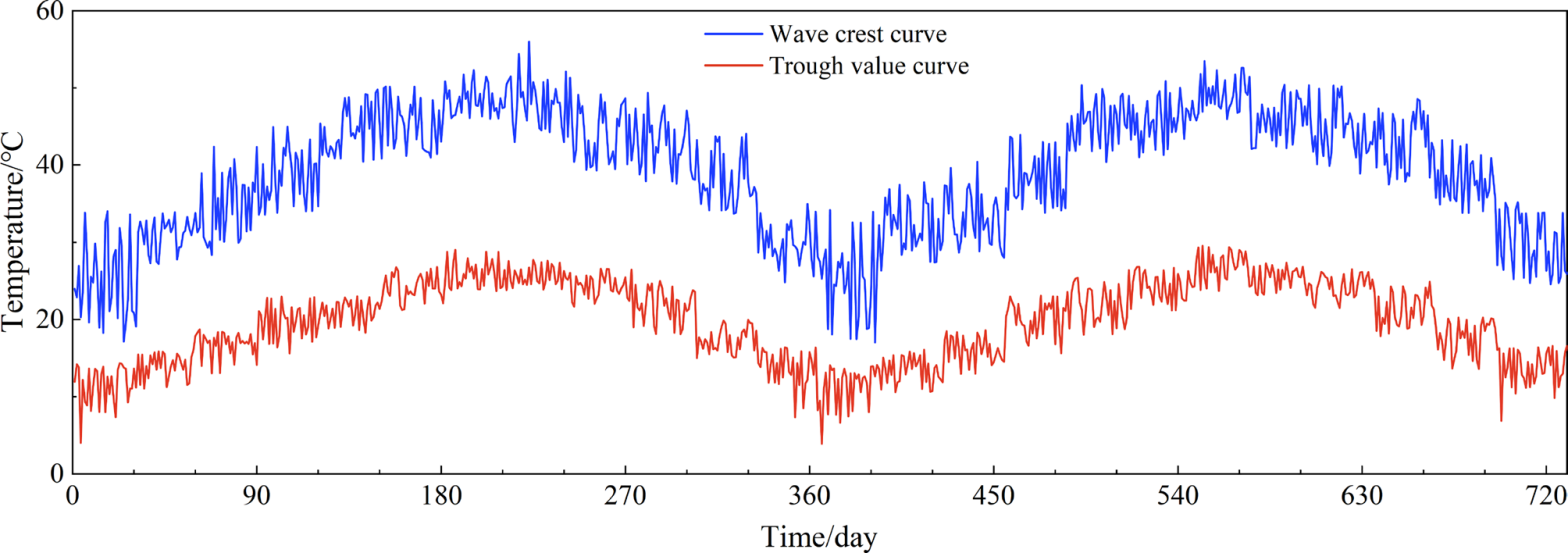
Simulated peak and trough of temperature field for 2 years.

**Figure 12:**
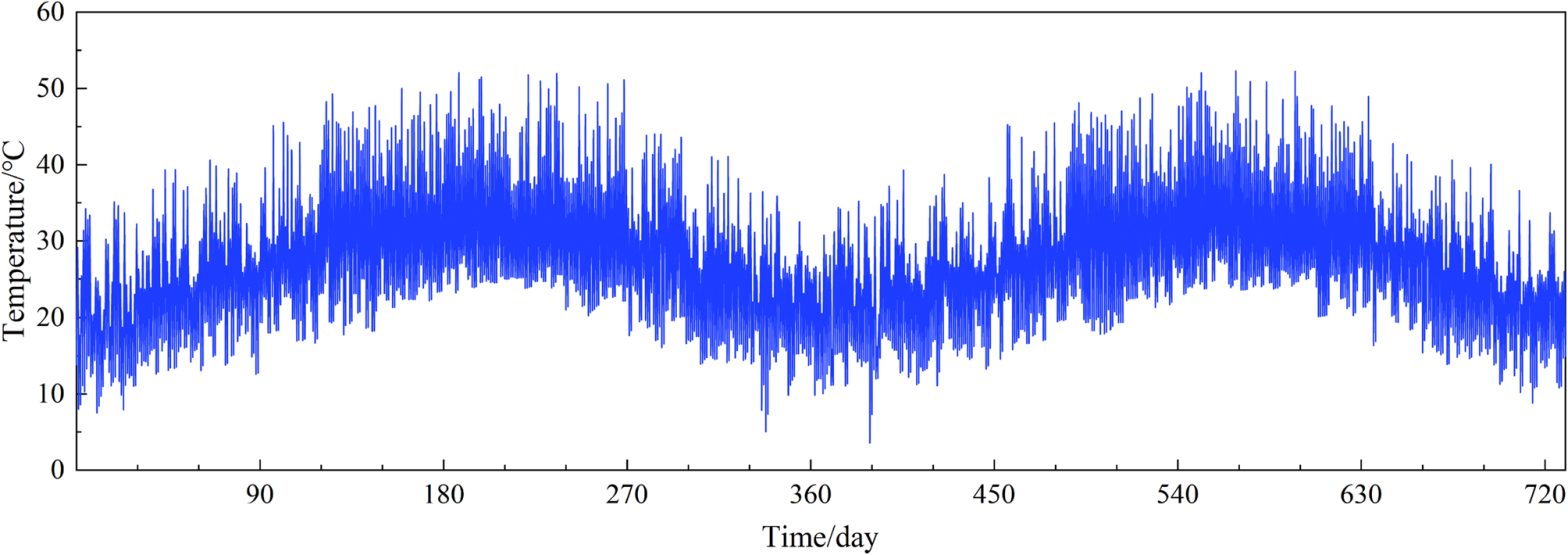
Simulated temperature field for 2 years.

Firstly, to calculate the monthly maximum peak and trough, minimum peak and trough by utilizing the monitoring temperature sample T2, then choosing two samples from the simulated *T*_*2,all*_ to construct the daily peak *T*_*peak*_ and trough *T*_*trough*_ for a certain day, satisfying *T*_*min,peak*_ < *T*_*peak*_ < *T*_*max,peak*_ and *T*_*min,trough*_ < *T*_*trough*_ < *T*_*max,trough*_, repeating this operation until all days have been assigned a pair of peak and trough. After the modification, the daily peak and trough of the simulated temperature field presented periodic variation already, 2 years’ data was shown in Fig. 11 as an example. At end, inserting the modified daily peak *T*_*peak*_ and trough *T*_*trough*_ into Eq. (7), the temperature sample simulation for one day could be eventually achieved, as shown in Fig. 12.

### Simulation of temperature difference sequences

**Figure 13:**
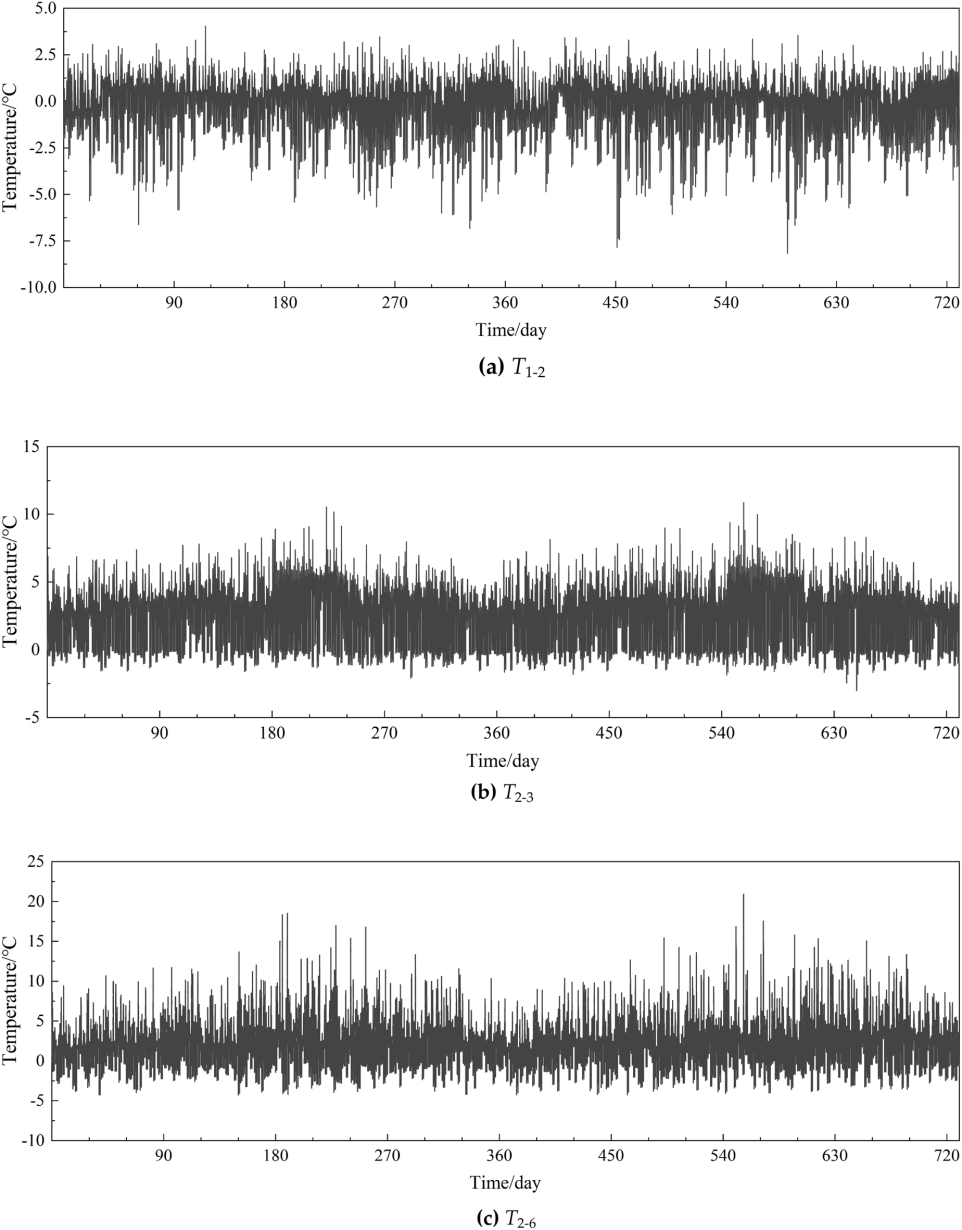
Simulated temperature difference sequences for 2 years.

**Figure 14:**
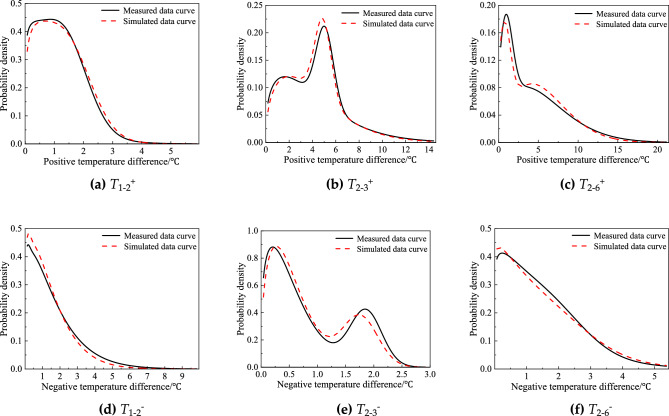
Comparison of PDFs of simulated temperature difference with observations.

For the simulation of temperature difference *T*_*1−2,all*_, *T*_*3−2,all*_ and *T*_*6−2,all*_ during the bridge life cycle, it could be fulfilled by repeating the simulation procedures of the benchmark point C2 mentioned above, to generate samples of *T*_*1−2*_, *T*_*3−2*_ and *T*_*6−2*_ with the same time scale. The simulated temperature difference sequences for 2 years were exhibited in Fig. 13. Furthermore, the PDFs of the simulated *T*_*1−2,all*_, *T*_*3−2,all*_ and *T*_*6−2,all*_ for one year were also investigated by means of mixture distribution model aforementioned, which were compared with the PDFs of all observations in 2021 in Fig. 14. The high similarity of the PDFs testified the availability of the proposed method.

## Conclusions

In this study, the temperature-induced effects on wide steel box girder was investigated based on 1-year monitoring data collected by the SHM system installed on a long-span suspension bridge located in South China. Specifically, the probability distribution characteristics and their statistics parameters of daily and yearly temperature observations were initially studied, moreover, the temperature field simulation method was further proposed for providing substantial data to implement structural safety estimation during bridge life cycle. The feasibility of the proposed method was verified through the field monitoring data. The following conclusions can be drawn from this study.

1. The temperature at each measuring point of the wide steel box girder cross-section changed on a daily basis due to the effects of sunlight. The top plate experienced a greater range of temperature variation compared to the bottom plate. The temperature fluctuation was nearly identical at the symmetrical measuring location, where the middle line of the large steel box beam section serves as the axis of symmetry.
2. Solar radiation made great contribution to the distributions of temperature field along the wide box girder. During the daytime on sunny days, notable vertical temperature gradients were observed, whose maxima reached to 21.39 °C and had exceeded the provision value of 20 °C specified in Chinese bridge design code. It also should be noted that considerable transverse temperature difference of 15.28 °C was inspected on the top plate of the box girder, which would produce non-negligible structural stress on the members.
3. Integrating the analyzed probability density functions with the exceedance probability method, representative values of temperature difference for various return periods were calculated. The transverse and vertical temperature differences for 50-year return period were 19.55 °C and 23.10 °C, respectively, closed to that of 24.0 °C in British BS5400-4.
4. The proposed simulation approach taking advantage of Latin hypercube sampler, was apply-cable in predicting temperature field for long-term condition, which was beneficial to structural evaluation of in-service bridges to ensure their serviceability and integrity in the life cycle.

Due to the conventional design of the steel box girder analyzed in the manuscript, the breadth was large and served as a reliable representation. The research findings could serve as a guide for the design of sections in steel box girders and for the reduction of fatigue damage resulting from temperature variations.

## Data Availability

The data presented in this study are available through email upon request to the corresponding author.
